# Event and Comment

**Published:** 1919-06

**Authors:** 


### DENTAL REGISTER

THE MAGAZINE OF DENTISTRY

Vol. LXXIII JUNE, 1919 No. 6

### EVENT AND COMMENT

**The Improvidence of Dentists.** We are reprinting in this issue from *Dental Facts,* a paper which seems to be an authentic exposition of the career of the average business man, as determined by a careful gathering of statistics by the American Bankers' Association and the insurance companies. The editor of *Dental Facts* submits it as a probable illustration of the career of the average dentist, although of course he can have no definite statistics to prove such a conclusion. It, however, is well worth reading most considerately by every young man who has selected dentistry as a profession. While there are undoubtedly some dentists who fail to save their earnings during the prosperous periods of their careers and so go through life living day by day and taking little or no precautions for the unproductive time when they must be dependent on the generosity of their children, friends, or possibly on some eleemosynary institution, the percentage of financial wrecks is probably not as high as is found in general business careers.

Dentists must live respectably and participate in most of the civic and social affairs of their town in order that they may justify their education and sphere of useful service. This very fact forces a dentist to provide very soon in his career for such expenses in addition to his ordinary personal needs. He is compelled to earn more and therefore exercises all his skill and faculties to enable him to meet such obligations as they appear. This necessarily stimulates frugality in living and spending, and generates a saving habit or plan. So that we believe dentists, as a class, will qualify more strongly with provident careers than the average business men. Dentists are often spoken of as bad business men and some are. Many make bad investments and lose their earnings because they have no adequate knowledge of sound investments or securities, and so they become the victims of dishonest promoters and reckless business men, but most dentists soon get wise to unfair business methods and seek legitimate lines of investment which do not tempt by promises of large gains in brief periods. These are so unusual that they, after a few bad experiences, lose their attractive characteristics. There should be no such disastrous results as are disclosed in this article to which we refer for the aged dentists. Only five of these one hundred business men left enough money to pay their own funeral expenses. This seems so incredible that we can hardly believe it, but the statistics are well credited from the most reliable sources. If there is any possibility that such results are probable for dentists it seems that we should begin to make definite preparations very early in our careers. Our code of ethics should be revised at an early date so that the question of providing for the unproductive period of life should be made one of its chief articles. Would it not be wise for the directors of our National Dental Relief Organization to take up a propaganda of calling to the attention of all dentists the absolute need of giving this matter attention at an early period of every professional man's career. No more important work can engage our attention.

